# Surgical Strategies for Ingrown Toenails: A Comprehensive Review of Techniques, Outcomes, and Advancements

**DOI:** 10.7759/cureus.52501

**Published:** 2024-01-18

**Authors:** Rao E Hassan, Luqman Khan, Syed Hussaini Shah, Hamid Naeem, Nazish Noor, Momina Iqbal, Faiza Dawood Khan, Zahir Rehman, Waheed Ahmad, Shafiq Tanveer, Arif Ullah Khan, Syed Hassnain Shah

**Affiliations:** 1 Orthopaedics and Trauma, Khyber Teaching Hospital Medical Teaching Institute (MTI), Peshawar, PAK; 2 Emergency Medicine, Russells Hall Hospital, Dudley, GBR; 3 Cardiac Surgery, Rehman Medical Institute, Peshawar, PAK; 4 Dermatology, Lady Reading Hospital Medical Teaching Institute (MTI), Peshawar, PAK; 5 Surgery, Ayub Teaching Hospital, Abbottabad, PAK; 6 General Surgery, Hayatabad Medical Complex Medical Teaching Institute (MTI), Peshawar, PAK; 7 Ophthalmology, Khyber Teaching Hospital Medical Teaching Institute (MTI), Peshawar, PAK; 8 Orthopaedics and Trauma, Pakistan Institute of Medical Sciences, Islamabad, PAK

**Keywords:** matricectomy, chemical matricectomy, super u procedure, vandenbos procedure, winograd technique, nail fold surgery, surgical management, podiatric procedures, onychocryptosis, ingrown toenails

## Abstract

Ingrown toenail (IGTN), known as onychocryptosis or unguis incarnatus, is a painful condition affecting the big toe, with symptoms including pain, inflammation, and infection. This review explores surgical options for IGTN, categorized into altering the nail plate or diminishing periungual tissues. Conservative treatments alleviate early-stage symptoms, while surgical interventions are reserved for severe cases. Various surgical techniques are discussed, such as the Winograd technique, Vandenbos procedure, chemical matricectomy, radiofrequency ablation, bipolar diathermy, carbon dioxide laser ablation, Zadik’s procedure, Howard-Dubois procedure, Super U procedure, Noël’s procedure, knot technique, and toenail paronychium flap. The choice of procedure depends on the severity and recurrence of IGTN.

## Introduction and background

Ingrown toenail (IGTN), also called onychocryptosis or unguis incarnatus, is a painful nail condition primarily affecting the big toe. The toenail’s edge compresses or penetrates the tissues of the lateral nail fold [1]. The term unguis incarnatus, which signifies an IGTN, describes the state of the ingrowth. In contrast, the alternate term for the condition, onychocryptosis, pertains to the coverage of the lateral and free edge of the toenail by the lateral nail fold [2,3]. Symptoms include pain, inflammation, and potential infections, with a 2.5-5% prevalence in teenagers and young adults [4]. Lifestyle changes and increased health awareness may contribute to IGTN. Diagnosis is straightforward, and treatment options range from conservative medical approaches to surgical interventions, depending on severity. This review explores the various surgical options for managing IGTN.

## Review

Pathogenesis

IGTN, caused by the toenail edge penetrating the skin, results in inflammation and possible infections. Improper nail plate trimming, wearing poorly fitting shoes, inadequate foot hygiene, malalignment of the matrix, abnormally long toes, hyperhidrosis, anatomical deformities, trauma, certain drugs, and genetic factors contribute to this condition. The common issue involves the lateral toenail part growing into the lateral nail fold, often due to improper trimming, leading to sharp regrowth. Factors such as tight footwear (high heels), tight socks, foot sweating, and diabetes mellitus exacerbate the problem. In such cases, actions such as cutting the lateral edges can perpetuate a harmful cycle [1]. Paronychia, marked by lateral embedding and granuloma formation causing IGTN, can arise as a side effect in antiretroviral agents, epidermal growth factor receptor inhibitors, and systemic retinoid treatments [5].

Various theories have been presented about the pathogenesis of IGTNs, sorting them by whether the problem arises from the nail itself or the surrounding soft tissues [6,7]. A hypothesis proposes that IGTNs develop due to lateral or distal nail pressure, exacerbated by poorly fitting footwear. Individuals with naturally curved nail plates are more susceptible. Although prevalent in adolescents and young adults because of increased foot perspiration, older individuals may confront persistent issues owing to diminished mobility or thickened toenails, imposing pressure on the skin [6].

Symptoms

IGTN presents with inflammation, swelling in the nail folds, and intense pain. Mozena’s classification system delineates five stages of IGTN, each revealing distinct symptoms and considerations for diagnosis and treatment. In the inflammatory stage (Stage I), individuals experience pain when pressure is applied to the lateral nail fold, mild edema, and erythema, emphasizing that the nail fold does not extend beyond the nail plate. Advancing to the abscess stage (Stage IIa), there is a noticeable escalation in pain, edema, erythema, hyperesthesia, and the potential for oozing or infection. In this stage, the nail fold extends over the nail plate, measuring less than 3 mm. The hypertrophic nail fold extending over the nail plate but measuring more than 3 mm is classified as Stage IIb. The hypertrophic stage (Stage III) is marked by the development of granulation tissue and persistent hypertrophy of the nail fold, which extensively covers the lateral nail plate. Progressing to the distal hypertrophic stage (Stage IV), a chronic deformity affects both lateral nail folds and the distal fold, resulting in the complete envelopment of the toenail by hypertrophic tissue [8]. This classification improves the understanding of IGTN progression, facilitating accurate diagnosis and the application of targeted treatment strategies.

Diagnosis

The diagnosis of IGTN is typically straightforward based on the patient’s medical history and clinical observations. Key indicators involve edematous lateral nail fold, often accompanied by discharge and the development of granulation tissue. The examination includes assessing the lateral edge of the nail to ascertain if it is growing beneath the skin, occasionally visualizing the penetration of the nail bed. Common signs encompass inflammation in the affected area, with apparent drainage serving as a clear signal of infection [9] In most cases, testing is unnecessary, but when a fungal infection is suspected, potassium hydroxide treatment and fungal culture may be conducted [10]. Various medical conditions, including cellulitis, osteomyelitis, foreign bodies, and tumors, can manifest with symptoms similar to IGTNs. If initial treatment proves ineffective, the use of X-rays and cultures may be considered to rule out these alternative conditions [11].

Complications

Paronychia, a common secondary infection resulting from IGTNs, can be caused by various pathogens such as *Staphylococcus *and superficial dermatophytes. Timely treatment of secondary infections associated with or following IGTNs is vital to prevent complications, including scarring of the nail fold and skin. In rare instances, there is a risk of cellulitis and osteomyelitis, posing an elevated threat of severe infections and amputations, particularly among individuals with diabetes [11]. Some individuals may have a genetic predisposition to recurrent IGTNs, significantly impacting their quality of life due to persistent pain and recurring infections.

Treatment

Managing IGTNs involves a range of approaches, each chosen based on factors such as the stage and severity of the condition, the surgeon’s expertise, and the patient’s treatment history. For mild-to-moderate cases with minimal pain, slight tissue hardening, and no signs of infection or abscess, conservative treatments are appropriate. Conversely, surgical procedures are typically reserved for severe cases marked by intense pain, inflammation, and the presence of infection or abscess.

The initial step in providing relief involves addressing symptoms, including the removal of the embedded spicule and meticulous cleaning of the area with an iodine or hydrogen peroxide solution to minimize infection risk. Additionally, patients can alleviate inflammation by gently massaging the affected area [12].

Conservative management

Opting for conservative and non-invasive treatments is the preferred approach to safeguard the lateral fold from injuries related to toenails. It is pivotal to alleviate pressure on the lateral fold and establish an unobstructed growth path for the toenail. This strategy is essential for both preventing and managing early-stage diseases while offering support in advanced treatment stages. Various methods are available to achieve this goal. Taping, the least invasive method, effectively pushes the lateral nail fold away when correctly applied during the early stages of the condition. Tamponing is suitable for all disease stages, involving the use of a fibrous compress dressing to create space between the lateral fold and the nail [13]. The dental floss method is a straightforward technique utilizing dental floss to separate the lateral fold from the edge of the nail, resembling tamponing. Protective tubes, longitudinally cut and inserted into the lateral fold, shield it from nail-induced pressure. Orthonyxial clips provide a non-invasive approach to reduce pressure on lateral nail edges, functioning similarly to orthodontic appliances. The gutter splint technique involves splinting the nail’s lateral edge with a vinyl intravenous infusion tube, secured with tape. Nail wiring achieves conservative results by creating holes at the distal end of the toenails and covering deformities with wires. Additional conservative approaches encompass using an acrylic artificial toenail, employing a slip tape-strap procedure, and utilizing a nail brace [4].

Surgical management

Moderate-to-severe instances of IGTN require surgical treatment. Moreover, in cases where initial efforts at non-invasive IGTN management prove ineffective, surgical procedures become the subsequent course of action. Surgical interventions can either serve as the primary option or be employed in response to a recurrence following unsuccessful initial surgical maneuvers. For all these techniques, digital blocks are sufficient for anesthesia of the nail bed and nail fold, and a tourniquet is applied at the metacarpophalangeal joint to obtain a bloodless field.

Various procedures can effectively manage IGTNs, classified into two main approaches, namely, diminishing periungual tissues or altering the nail plate. Treatment modalities such as simple nail avulsion, wedge resection with or without coagulation (Winograd’s procedure), matricectomy using chemical agents, carbon dioxide laser or electrocautery, and Zadik’s procedure primarily aim at correcting the nail plate. In contrast, Vandenbos, Howard-Dubois, Noël’s procedure, and Super U technique are efficient in excising surplus soft tissue, specifically addressing the hypertrophic nail folds [14].

Winograd technique (wedge resection of the toenail and nail bed)

**Figure 1:**
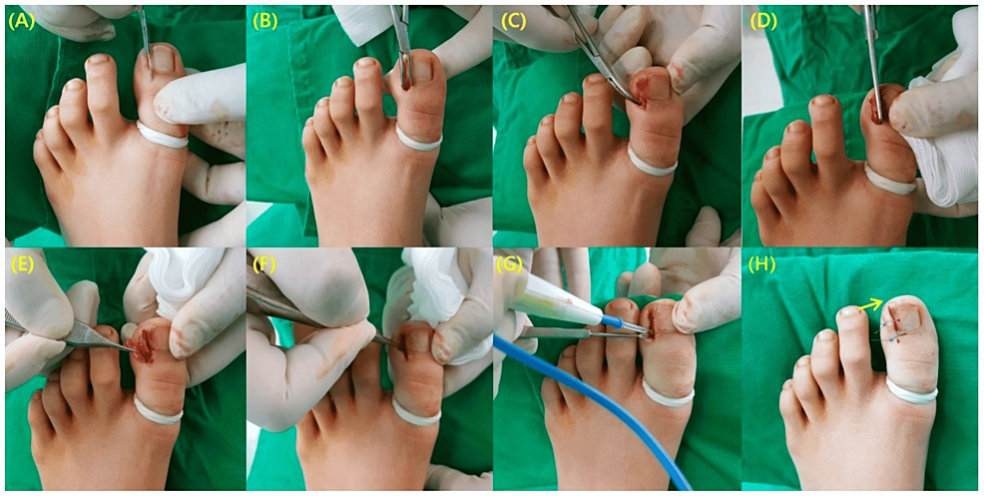
Representation of modified Winograd technique. (A) Creation of a 6-8 mm incision at the eponychium. (B) Application of blunt dissection to separate the soft tissue from the ingrown part. (C) Exposure of the overgrown nail plate. (D) Partial excision of the nail plate. (E) Excised portion of the nail plate. (F) Meticulous curettage of the germinal matrix and nail bed. (G) Additional destruction of the germinal matrix using electrocautery. (H) Incorporation of one nylon suture to securely attach the resected eponychium. Notably, in instances of an unstable nail plate, an extra distal suture is applied, indicated by the arrow. From Kim et al. (2021) [17]. This article is an open-access article distributed under the terms and conditions of the Creative Commons Attribution (CC BY) license (http://creativecommons.org/licenses/by/4.0/), and no changes were made to the original material.

Excision entails the removal of a wedge, encompassing the nail portion up to the germinal matrix and the hypertrophied soft tissue up to the bone level. Following this, the remaining soft tissue is sutured to the nail [15]. Post-procedure, a sterile dressing is applied, accompanied by compression bandages. Khan et al. conducted a study on 29 toenails. They concluded that it is a straightforward and safe procedure, with recurrence rates and complications deemed acceptable. However, it is vital to communicate to patients, especially females, the potential for recurrence and cosmetic alterations in their toenails [16]. Kim et al. modified the Winograd technique utilizing a surgical curette. With the help of a curette, precision is employed to remove and eliminate the germinal matrix and nail bed. The excision of granulation tissue at the lateral edge of the nail plate removes the need for resecting the lateral nail fold. Subsequently, the germinal matrix and nail bed undergo thorough coagulation using electrocautery. The original method, combined with electrocauterization and excluding wedge resection, proves to be a minimally invasive, straightforward, swift, and highly effective approach for treating IGTNs, leading to heightened patient satisfaction (Figure 1) [17].

Vandenbos procedure

**Figure 2:**
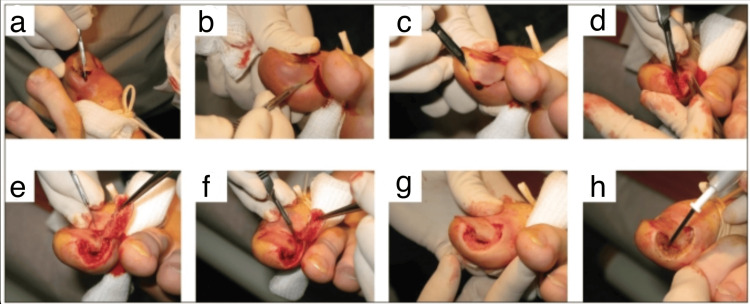
Vandenbos approach to treating ingrown toenails. (a) The toe is sterilized with an iodine solution, and ring block anesthesia is administered using 2% xylocaine without epinephrine. A tight elastic tourniquet is placed at the toe’s base to ensure a clear surgical field. (b-d) An initial incision is made approximately 5–10 mm proximal to the nail’s base and 3–5 mm from the lateral edge, encompassing the proximal nail fold while preserving the nail matrix. The lateral nail fold is then removed with a lateral elliptical sweep, extending distally to include all affected granulation tissue and adjacent soft tissues. (e, f) The skin and subcutaneous tissues at the nail’s edge are completely excised, occasionally exposing a portion of the distal phalanx. (g) Following the thorough removal of soft-tissue nail folds, the intact nail and preserved nail matrix become visible. (h) Achieving hemostasis with electrocautery, the wound is left open for secondary intention closure, and gauze dressings are applied (h). Reproduced from Chapeskie and Kovac (2010) [21]. The article is used here for non-commercial educational purposes, as allowed by the publisher at https://www.canjsurg.ca/page/copyright-permission. Furthermore, permission was also granted from the corresponding author. © 2010 Association Médicale Canadienne.

Starting with a 5 mm proximal incision, the integrity of the nail bed is meticulously preserved. Protective measures are initiated approximately 3 mm from the lateral edge of the nail base, safeguarding the nail matrix. An elliptical incision extends laterally and distally, removing all granulation tissue and the skin surrounding the nail fold. This removal is crucial for comprehensive extraction, allowing for the creation of a 1.5 × 3 cm defect. Pressure is applied post-tourniquet removal and bleeding foci are cauterized to minimize postoperative bleeding. Special attention is devoted to preventing damage to the nail matrix. The wound is left open, and a generous amount of gauze is applied to counter potential postoperative leakage-induced bleeding (Figure 2) [18]. Karacan et al. demonstrated that the Vandenbos method demonstrates lower recurrence rates, heightened patient satisfaction, and superior cosmetic outcomes in comparison to the Winograd technique [19]. Nasr et al. applied this technique to children and adolescents, discovering that the procedure is associated with a minimal recurrence rate in this population experiencing IGTNs. Patients reported excellent recovery times, low complication rates, positive functional outcomes, and high satisfaction levels [20]. Chapeskie et al. introduced a modified surgical approach that focused on removing granulation tissue around the nail fold while preserving both the nail and nail matrix. This modification showed no recurrences, resulted in excellent cosmetic outcomes, and achieved high levels of patient satisfaction [21].

Chemical matricectomy after partial nail avulsion

In this technique, a partial nail avulsion is performed, involving the extraction of the embedded portion from the nail fold along with a segment of the normal lateral nail. Subsequently, a chemical agent, typically phenol, is applied for matricectomy, yielding positive outcomes and a low risk of recurrence. It is crucial to note that the use of phenol may result in significant damage to the surrounding tissue, potentially causing drainage issues and delayed wound healing [22]. To mitigate these concerns, a safer approach involves applying phenol for a brief duration of one minute, followed by rinsing with 70% isopropyl alcohol to neutralize its effects. This method is deemed sufficient for the elimination of the germinal matrix [23]. Moreover, 10% sodium hydroxide is equally effective as phenol, inducing liquefactive necrosis via alkali burning. This reduces postoperative drainage and accelerates healing. However, caution is essential to avoid prolonged application of strong alkali, preventing excessive tissue damage due to gradual liquefactive necrosis [24]. Chemical matricectomy with 90% trichloroacetic acid is employed after partial nail avulsion, providing benefits with minimal postoperative complications. This approach is secure, straightforward, and effective, demonstrating few postoperative issues and a high success rate [25].

Radiofrequency ablation

It is a relatively less commonly employed technique. It was investigated by Singal et al. in a study involving eight adult patients with Stage 2 IGTNs unresponsive to conservative treatments. The procedure includes partial nail avulsion followed by applying an electrode to the matrix over the lateral horn of the nail for three to five seconds, repeated two to three times. The average healing time was 7.5 days. By the fourth day, oozing ceased, and there was a substantial improvement in erythema, pain, and edema. No postoperative complications occurred, and there were no recurrences noted during the three- to six-month follow-ups [26].

Bipolar diathermy

Similar to radiofrequency ablation, this method includes the wedge excision of the ingrown nail and the use of bipolar diathermy on the nail bed. Farrelly et al. investigated this approach, conducting 353 procedures involving 302 patients. The re-operation rate for recurrence was 9.9%, favorably comparable with alternative techniques [27].

Carbon dioxide laser ablation

After partial nail avulsion, carbon dioxide laser matricectomy is frequently recommended, exhibiting recurrence rates comparable to other techniques (2-6%) [28,29]. Its benefits include minimal tissue damage within the cauterized area, effective bleeding control, pain relief, reduced postoperative infections, and a lower recurrence risk. Laser treatment for IGTNs is recognized for its coagulation properties and precise incision margins during the surgical procedure [29]. Drawbacks encompass toenail disfigurement and an extended healing period [30].

Zadik’s procedure

**Figure 3:**
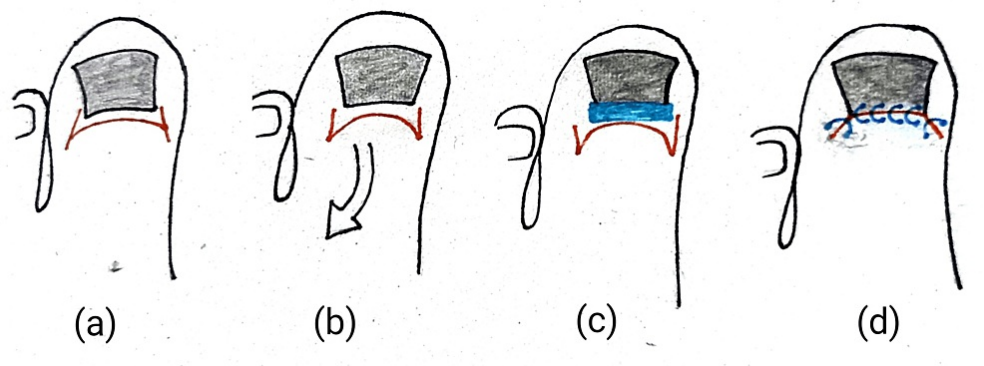
Steps of Zadik’s procedure after the removal of the nail. (a) A classic H incision is made, with the transverse cut angled at approximately 90 degrees to expose the proximal matrix. (b) Extending the incision both medially and laterally reveals an oval germinal tissue area as the flap is retracted proximally. (c) A rectangular section of tissue is then excised down to the underlying phalanx, paying specific attention to the lateral horns of the matrix to minimize spicule formation. (d) Closing the wound entails advancing the proximal nail fold flap to the nail bed, securing it with simple interrupted polypropylene sutures. Minimal tension is applied during this process to preserve flap viability. Self-generated depiction of Zadik’s procedure.

In this traditional technique, the nail plate undergoes separation from the nail bed and surrounding tissues through blunt dissection, leading to complete removal. The nail bed is then prepared with povidone-iodine to ready the epithelium for incision. A traditional H incision is executed with a transverse cut angled at about 90 degrees to expose the proximal matrix. Drawing the flap back proximally reveals an oval area of germinal tissue, extending both medially and laterally. To minimize spicule formation, a rectangular section of tissue is carefully excised down to the underlying phalanx, with a particular emphasis on the lateral horns of the matrix. The precise removal of the germinal matrix is ensured using a small curette. To close the wound, the proximal nail fold flap is advanced to the nail bed, secured with simple interrupted polypropylene sutures, applying minimal tension to preserve flap viability (Figure 3) [31,32]. This procedure is now less commonly employed due to the increased risk of nail spicule regrowth resulting from incomplete matricectomy, high recurrence rates, disfigurement due to the narrowing of the nail plate, and postoperative infections in the nail bed [32,33].

Modified Howard-Dubois procedure

**Figure 4:**
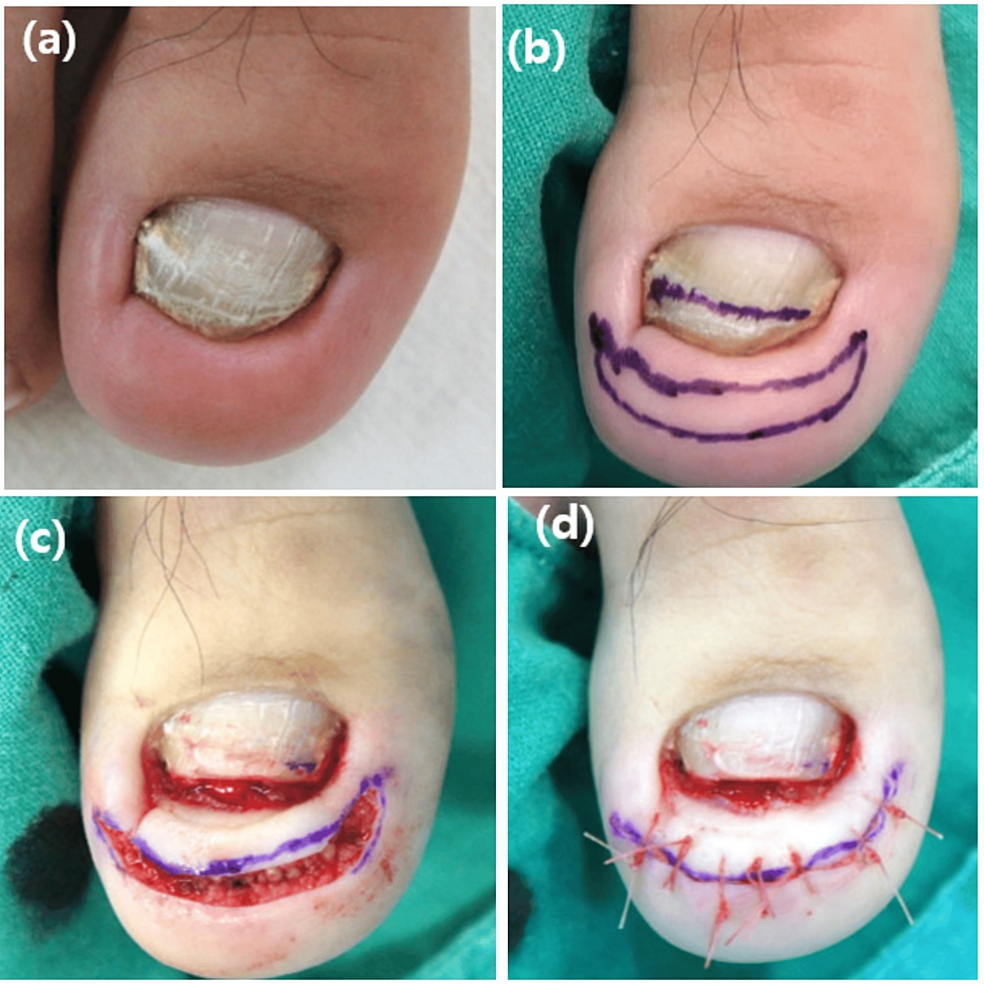
Illustration of the modified Howard-Dubois procedure. (a) Transverse rim observed at the distal edge of the toenail, accompanied by nail shortening and discoloration. (b) The incision line is delineated with a 7 mm width crescent shape running parallel to the distal and lateral nail folds around the toe tip, and a mark is placed on the distal third of the toenail. (c) Wedge-shaped removal of sufficient soft tissue is performed, along with the excision of the distal third of the toenail and curettage of the nail bed. (d) Sutures are utilized to pull down the thickened distal pulp (d). From Ha et al. (2023) [36]. This is an open-access article distributed under the terms of the Creative Commons Attribution-Non Commercial-Share Alike 4.0 License (http://creativecommons.org/licenses/by/4.0/), which allows others to remix, transform, and build upon the work non-commercially, as long as the author is credited and the new creations are licensed under the identical terms. No changes were made to the original material.

It is considered for mild-to-moderate cases [34]. Howard-Dubois in 1974 detailed a fish-mouth incision encircling the tip of the affected toe along the nail groove. It is crucial to protect a slim wide strip of skin beneath the lateral nail grooves and toe tip. Another incision is made adjacent to the initial one, creating a wedge of skin and subcutaneous tissue for removal. The width of the crescent is determined by the extent of hypertrophied granulation tissue or the depth of the buried nail plate [35]. In the subsequent stages of the Howard-Dubois technique, additional refinements were incorporated [14]. The periosteum dissector is used to separate the nail bed from the distal phalanx. A bone rongeur is then used to remove part of the dorsal section of the phalanx to flatten its surface, an important step in reducing the curvature of the nail bed after wound closure. Subsequently, the edges are brought together to achieve primary intention closure (Figure 4) [36]. Utilizing this method offers several benefits, including the elimination of recurrence, a quicker return to work, prevention of nail plate narrowing, symptom relief, increased patient satisfaction, and improved cosmetic appearance. Conversely, the drawbacks are associated with the intricate nature of the procedure and the potential for postoperative pain [14,36].

Super U procedure

**Figure 5:**
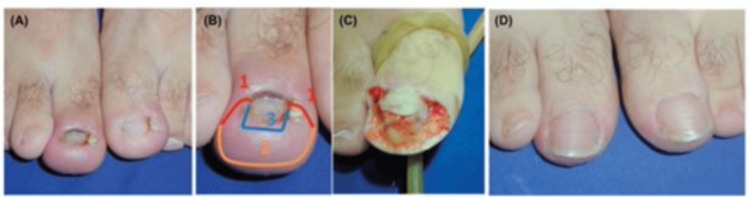
Demonstration of the Super U technique. (A) Severe ingrown toenail with nail deformity. (B) Three incision lines to be employed. (C) Intraoperatively after the removal of lateral and distal folds. (D) After eight months. From Rosa et al. (2015) [37]. Permission was obtained from Wolters Kluwer Health, Inc. No changes were made to the original material.

Reserved for severe cases of IGTNs [34], the technique involves two parallel U-shaped incisions to remove hypertrophic tissue. The initial bilateral incision, spanning from the cuticle to the outer edge of the hypertrophic tissue, is created perpendicular to the proximal nail fold. A second incision, in line with the bilateral distal lateral fold, forms a large U shape. A parallel U-shaped incision is then executed just beneath the initial U, aiding in the removal of hypertrophic tissue positioned between the two U-shaped lines. Fat tissue is preserved on the lateral folds, omitting the distal fold. Hemostasis is secured and healing occurs through secondary intention [37,38]. In their study, Correa et al. found that the Super U technique, preserving the entire nail and adapting the soft tissues of the big toe to the nail, significantly improves the cosmetic outcome compared to matricectomy (Figure 5) [38]. While considered the primary treatment for severe and recurrent cases, this approach has drawbacks, including the need for intensive nursing supervision, typically lasting five to seven weeks and contributing to prolonged morbidity [34,38].

Noël’s procedure

**Figure 6:**
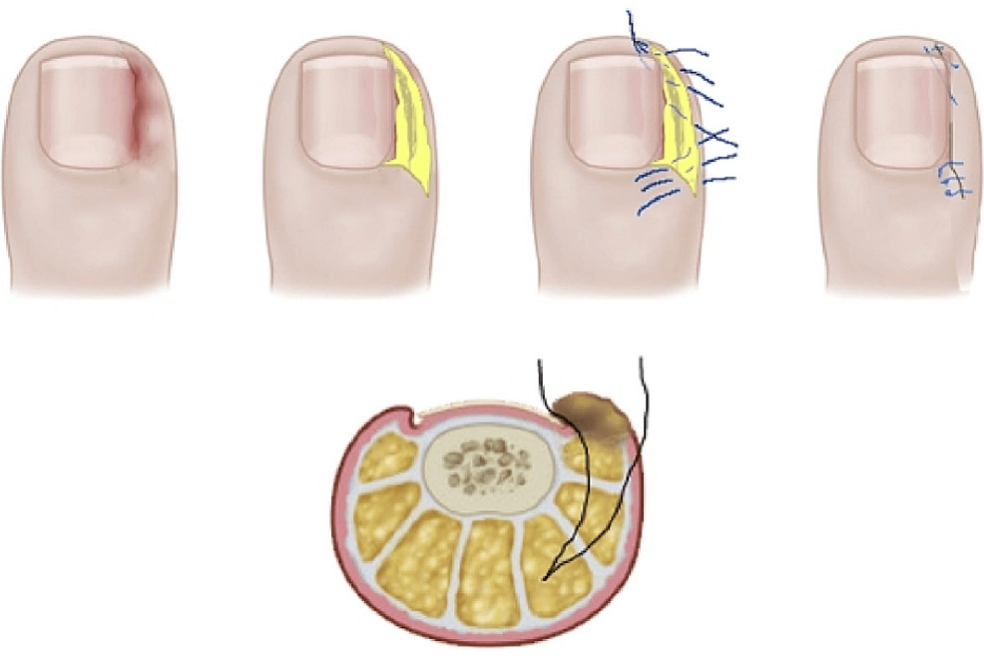
Dąbrowski’s surgical approach for addressing an ingrown toenail while preserving both the nail and matrix. From Dąbrowski and Litowińska (2020) [40]. This is an open-access article distributed under the terms of the Creative Commons CC-BY license, which permits unrestricted use, distribution, and reproduction in any medium, provided the original work is properly cited. No changes were made to the original material.

In 2008, Noël introduced a new technique for surgically treating IGTNs without matricectomy [39]. Subsequently, Dąbrowski et al. further refined this approach, achieving superior outcomes [40]. The procedure involves the excision of a wedge-shaped ellipse of soft tissue, encompassing fibrotic and granulation tissue, from both sides of the nail. Incision lines, aligned parallel to the rear of the nail, extend along the lateral borders of the nail plate, reaching about 4-6 mm distally. The extent of the semi-elliptical lateral cut is dictated by the size of the inflammatory lesion. Deep incisions are executed to remove a significant amount of soft tissue, and the wound is primarily closed using non-absorbable suture, preferably polypropylene [39]. Dąbrowski et al. modified this technique by introducing a subungual suture in the middle of the lateral wound margin, threading it through subcutaneous tissues, and ending it distal to the hyponychium. Additional sutures in the distal direction ensure the lateral wound edge is drawn beneath the nail. Proximal sutures approach the wound edges. Importantly, no nail avulsion is performed and the nail bed and matrix are prevented from any injury (Figure 6) [40]. Research findings indicate a decrease in recurrences and heightened levels of patient satisfaction with both the original technique and its suggested modifications [40,41].

Knot technique

**Figure 7:**
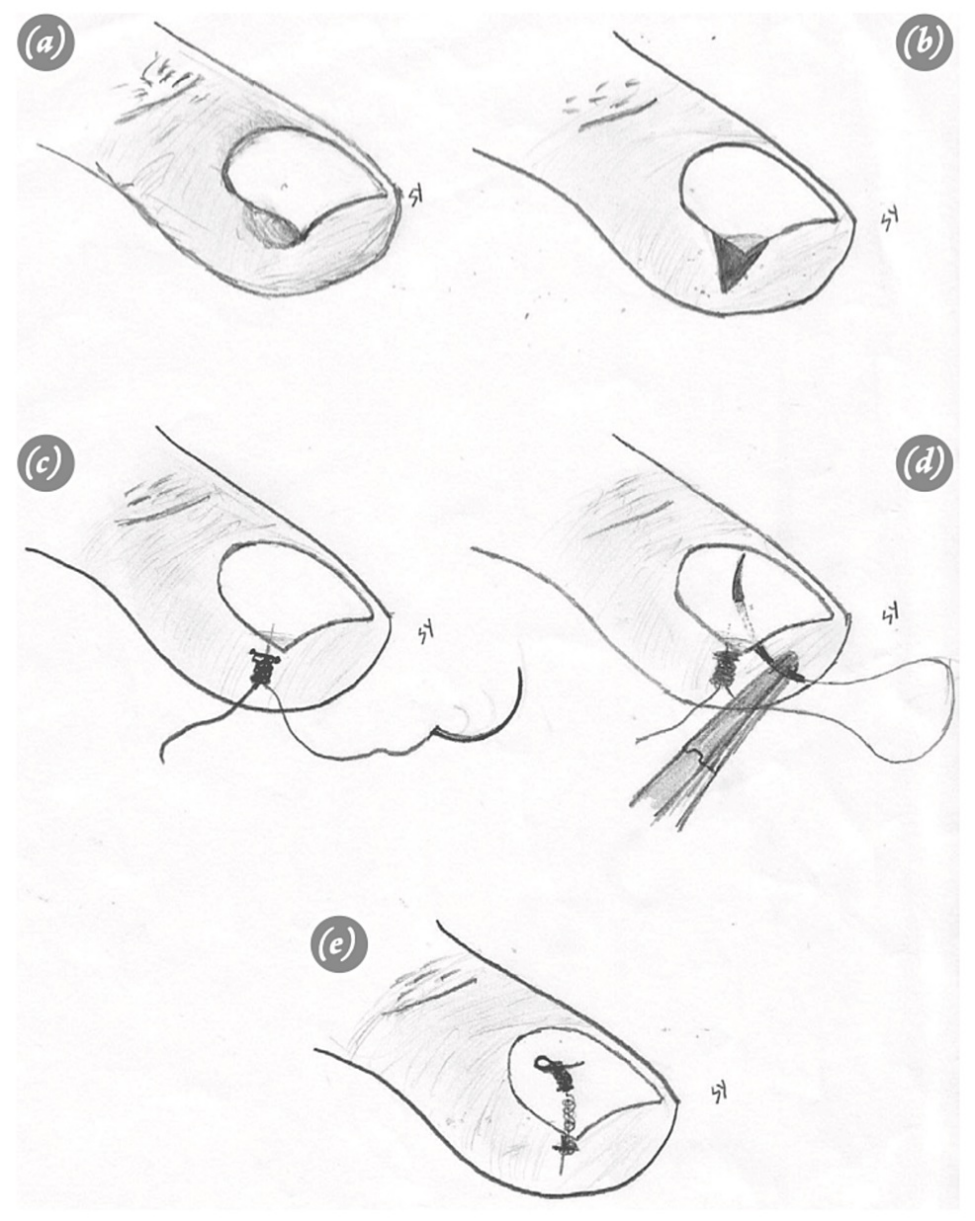
Demonstration of the knot technique. (a) Display of an ingrown nail. (b) Excision of the upper and lower soft tissues surrounding the nail through a wedge. (c) Tying 8–10 knots without cutting the stitches beneath the nail. (d) Inserting the needle inside the nail, tying a knot underneath, and subsequently tying another knot above the nail without cutting the suture. (e) Postoperative representation of the nail. From İnce et al. (2015) [43]. This article is an open-access article distributed under the terms and conditions of the Creative Commons Attribution (CC BY) license (https://creativecommons.org/licenses/by-nc/4.0/). No changes were made to the original material.

A wedge excision is conducted on the soft tissues surrounding the nail, both above and below. Following this, the wound margins are sutured with non-absorbable suture, tying approximately 8-10 knots without cutting the stitches beneath the nail. These knots serve to anchor the soft tissue and maintain the elevation of the nail edge [42]. In a follow-up study by Ince et al., the knot technique was compared to the Winograd method, highlighting that this technique serves as a straightforward and efficient approach for treating ingrown nails. This method is linked to a lower complication rate and shorter surgical durations (Figure 7) [43].

Toenail paronychium flap

**Figure 8:**
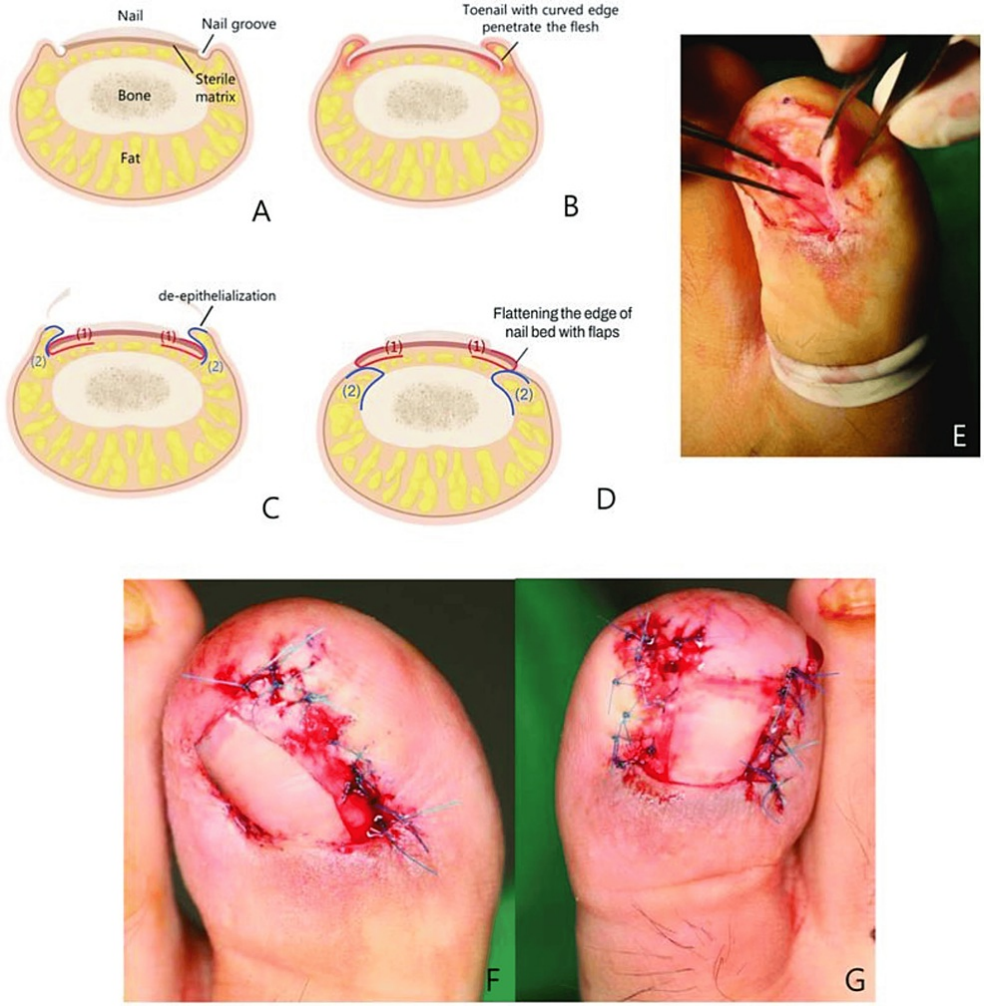
Demonstration of the paronychium flap technique. (A) Depicting a normal toenail and (B) an ingrown toenail. (C) Conducting de-epithelialization of lateral nail folds, raising flaps of the paronychium fat pad (2) and nail bed flaps (1). (D) The detached paronychium fat pad flap (2) is secured to the periosteum. Following this, nail bed flaps (1) are positioned over the paronychium fat pad flaps (2) and stitched to the paronychium skin. (E) Intraoperative images capture the elevation of nail bed flaps and paronychium skin flaps over the paronychium fat pad. (F, G) The illustration displays the suturing of nail bed flaps to the paronychium skin. From Ahn et al. (2023) [33]. This is an open-access article published by Thieme under the terms of the Creative Commons Attribution License, permitting unrestricted use, distribution, and reproduction so long as the original work is properly cited. (https://creativecommons.org/licenses/by/4.0/)/. No changes were made to the original material.

In this newer approach, granulation tissue is removed, and the lateral nail folds undergo de-epithelialization. The incision is precisely made at the lateral corner of the nail bed, where the lateral nail fold meets the nail plate. Extending it up to the proximal nail bed ensures complete exposure of the lateral matrix’s horn. Blunt dissection follows to separate the ingrown nail from the nail bed. A segment of the nail plate containing the ingrowth is excised, and the lateral matrix’s horn is extracted. Sharp dissection at the lateral edge is then performed to detach the nail bed from the periosteum beneath. The fat pad of the paronychium is isolated from the skin flap and secured to the periosteum using small absorbable sutures. The nail bed flap is subsequently positioned over the paronychium fat pad and sutured to the paronychium skin flap using a combination of small absorbable and non-absorbable sutures. In a documented case, this technique resulted in a permanent cure for a patient who had previously undergone multiple conservative and six operative interventions (Figure 8) [33].

In summary, procedures that involve altering the nail plate are typically the primary interventions. Recurrent surgery is more likely after plain avulsion or wedge avulsion without phenol application. Conversely, excision of the nail bed and subsequent application of phenol demonstrates the lowest recurrence rate [44]. Procedures involving the degloving of periungual tissues are reserved as a last resort for severe, recurrent, and deformed presentations. In addressing chronic IGTN characterized by hypertrophic lateral walls, Richert recommends employing the Vandenbos technique, Noël’s approach, the Super U method, and the Howard-Dubois procedure [45]. The summary of all procedures in this review is presented in Table 1.

**Table 1:** Summary of surgical techniques employed for ingrown toenails. NaOH: sodium hydroxide; TCA: trichloroacetic acid

Technique	Description	Advantages	Disadvantages	Indications	References
Winograd (wedge resection)	Removal of the nail wedge and nail bed + hypertrophied tissue. Soft tissue sutured to the nail	Simple, safe, early recovery	Cosmetic alterations, recurrence potential (12%)	First-line option for mild-to-moderate cases with limited involvement of the nail bed	[15,16]
Winograd modified (curette and electrocautery)	Precise curette removal of the germinal matrix and nail bed. Granulation tissue incision with scalpel, followed by electrocautery ablation. No wedge resection	Minimally invasive, quick, effective, high patient satisfaction, low recurrence rates	Cosmetic alterations	Preferred for precise germinal matrix removal and minimal tissue damage	[17]
Vandenbos	Elliptical incision preserving the nail bed and matrix. Removal of the skin, granulation tissue, and edge beneath the nail tip. Creation of skin/soft tissue defect. Secondary tissue healing	Low recurrence rate (nearly no recurrence), better cosmetics, high patient satisfaction	Prolonged recovery time, late return to work	Preferred for moderate-to-severe cases with greater soft tissue involvement	[18-21]
Chemical matricectomy after partial nail avulsion	Partial nail removal + chemical agent (phenol, NaOH, TCA) applied to destroy the germinal matrix	Low recurrence rate (Phenol 11%, NaOH ~5%, TCA <5%), effective	Tissue damage, drainage issues, delayed healing	Phenol for moderate cases with no prior surgeries, and NaOH and TCA are alternative to phenol in cases requiring safer options	[22-25]
Radiofrequency ablation	Partial nail removal + electrode applied to matrix for 3–5 seconds (repeated)	Fast healing, no complications, no recurrences on follow-up	Less common, limited research	An option for resistant cases unresponsive to conservative treatment	[26]
Bipolar diathermy	Wedge excision + bipolar diathermy on the nail bed	Low re-operation rate (9.9%), comparable to other techniques	Less common, limited research	Moderate-to-severe cases with significant soft tissue involvement	[27]
Carbon dioxide laser ablation	Partial nail removal + CO_2_ laser on matrix	Minimal tissue damage, effective bleeding control, pain relief, reduced infections, low recurrence risk (5%)	Toenail disfigurement, extended healing period	Considered for mild-to-moderate cases requiring precise ablation and pain relief	[28-30]
Zadik’s procedure	Complete nail removal + H incision to expose and excise the germinal matrix	Traditional technique, less commonly used now	High recurrence rate (16–28%), nail spicule regrowth, nail plate narrowing, disfigurement, postoperative pain and infections	Obsolete now, no clear indication of performing nowadays	[31-33]
Modified Howard–Dubois	Fish-mouth incision + wedge of skin/soft tissue removal + bone rongeur to flatten phalanx surface	Eliminates recurrence, quick return to work, prevents nail narrowing, symptom relief, high satisfaction, improved cosmetics	Intricate procedure, potential for postoperative pain and bleeding	Preferred for mild-to-moderate cases with distal and deep nail ingrowth	[34-36]
Super U	Two parallel U-shaped incisions to remove hypertrophic tissue and preserve fat tissue on lateral folds	Improves cosmetic outcomes compared to matricectomy, primary treatment for severe/recurrent cases, low recurrence rate (<1 and 2%)	Intensive nursing supervision (5–7 weeks), prolonged morbidity	Severe or recurrent cases with extensive soft tissue involvement and prior failed procedures	[34-38]
Noël/Dąbrowski	Wedge-shaped ellipse removal of fibrotic/granulation tissue + subungual suture	Decreased recurrences (Dąbrowski: 1.8%), high patient satisfaction	Prolonged duration of symptoms (up to one year)	Alternative for moderate-to-severe cases requiring minimal nail avulsion and preserving the matrix	[39-41]
Knot technique	Wedge excision + wound margin sutures tied 8–10 times beneath the nail, pushing down soft tissue and lifting nail	Straightforward, efficient, lower complication rate, shorter surgical duration	Cannot be employed for individuals who prefer to avoid having multiple knots on their nail for about a month, recurrence potential (11%)	Option for mild-to-moderate cases with easily accessible ingrowth and minimal involvement of the nail bed	[42,43]
Toenail paronychium flap	Granulation tissue removal + lateral nail fold de-epithelialization + flap creation and anchoring	Permanent cure for challenging cases, successful after multiple failed interventions	Requires further research, limited data	May be considered for challenging cases with prior failed surgeries, but needs more research	[33]

## Conclusions

In managing IGTNs, a spectrum of surgical interventions is available, ranging from altering the nail plate to diminishing periungual tissues. Conservative measures prove effective for mild cases, while severe instances necessitate surgical procedures. The selection of a specific technique depends on factors such as severity, recurrence, and patient history. The Winograd technique and Vandenbos procedure are notable for their effectiveness and low recurrence rates. Chemical matricectomy, radiofrequency ablation, and carbon dioxide laser ablation offer alternatives with distinct benefits. Each procedure has its advantages and drawbacks, emphasizing the need for personalized treatment plans. This review underscores the importance of accurate diagnosis and tailored interventions for optimal outcomes in managing IGTN.
